# Uniformity in Reprints

**Published:** 1889-10

**Authors:** 


UNIFORMITY IN REPRINTS.—The Pittsburgh *Medical Review,* acting upon the hint of Dr. Charles Everett Warren in the Boston *Medical and Surgical Journal,* and seconded by the St. Louis *Medical and Surgical Journal,* has taken up this important question, from the practical standpoint, in its September issue. It gives a table of column measurements of eighteen double column, and twenty-three single column journals, and arrives at the conclusion, from their study, that two regular sizes should be adopted, to secure absolute uniformity of publication.

We have experienced the difficulties that are complained of by the author and journals named, and have given considerable thought to a solution thereof. While we recognize the considerable cogency and force with which the editor of the *Review* states his argument, we are yet of the opinion that it would be better to adopt one standard, instead of two, for all reprint publications.

This could easily be done by adopting the standard octavo as the size for *all* reprints. This, it is true, would leave wide margins in those of the double column journals, for they would print but one column on a page; but they look well so printed, and the wide margins are convenient for annotations. We have even seen the narrow columns of a newspaper so reprinted, and were impressed with the beauty of the work. Perhaps such magazines as the *University* and the *North American Practitioner* would be willing to reduce to the standard octavo, of which the *American Journal of the Medical Sciences* may be cited as a type, for the sake of bringing about this much-desired uniformity.

